# Misfolding at the synapse: A role in amyotrophic lateral sclerosis pathogenesis?

**DOI:** 10.3389/fnmol.2022.997661

**Published:** 2022-09-09

**Authors:** Jeremy S. Lum, Justin J. Yerbury

**Affiliations:** ^1^Illawarra Health and Medical Research Institute, Wollongong, NSW, Australia; ^2^Molecular Horizons and School of Chemistry and Molecular Bioscience, University of Wollongong, Wollongong, NSW, Australia

**Keywords:** proteostasis, axo-synaptic compartment, protein misfolding, synapse, amyotrophic lateral sclerosis

## Abstract

A growing wave of evidence has placed the concept of protein homeostasis at the center of the pathogenesis of amyotrophic lateral sclerosis (ALS). This is due primarily to the presence of pathological transactive response DNA-binding protein (TDP-43), fused in sarcoma (FUS) or *superoxide dismutase-1* (*SOD1*) inclusions within motor neurons of ALS postmortem tissue. However, the earliest pathological alterations associated with ALS occur to the structure and function of the synapse, prior to motor neuron loss. Recent evidence demonstrates the pathological accumulation of ALS-associated proteins (TDP-43, FUS, C9orf72-associated di-peptide repeats and SOD1) within the axo-synaptic compartment of motor neurons. In this review, we discuss this recent evidence and how axo-synaptic proteome dyshomeostasis may contribute to synaptic dysfunction in ALS.

## Introduction

Proteome homeostasis (proteostasis) refers to the controlled maintenance of each protein in the proteome in its precise conformation, concentration and location for each cell to carry out its function. Maintaining proteostasis requires various biological mechanisms to regulate the synthesis, degradation, (re)-folding and trafficking of the proteome. The mechanisms required to maintain proteostasis are finely tuned in all cell types. However, an inability of cells to maintain proteostasis can lead to the accumulation of misfolded proteins, particularly within uncharacteristic cellular compartments, leading to cellular dysfunction and death.

Neurons are particularly susceptible to proteostasis network dysfunction compared to other cell types (Kundra et al., 2020). An inability for the neuron to maintain its proteome leads to the accumulation of misfolded/aggregating proteins, subsequently causing proteome collapse and the formation of inclusions (Knowles et al., 2014). An accumulation of misfolded/aggregated proteins and inclusions can be observed within distinct neuronal cell types and anatomical regions in several neurodegenerative disorders including Alzheimer’s Parkinson’s, Huntington’s disease (Yerbury et al., 2016). Similarly, amyotrophic lateral sclerosis (ALS), which causes the progressive loss of upper and lower motor neurons also exhibits the accumulation of misfolded proteins and inclusions within motor neurons (Leigh et al., 1991; Ticozzi et al., 2010). ALS cases are generally categorized as either familial (family history of genetic mutation) or sporadic (no family history). Despite the origin of disease, a common characteristic is the presence of proteinaceous inclusions (Bunina bodies, basophilic inclusions, skein-like inclusions and hyaline inclusions) within motor neurons and neighboring cells. The particular inclusion type and anatomical region observed can vary based on the presence or absence of ALS mutation (Saberi et al., 2015). However, proteostasis dysfunction that allows aggregates to proceed into inclusions across all forms of indicate this is a common mechanism underlying ALS.

Whilst, the presence of inclusions are observed in post-mortem tissue, on the other end of the disease timeline, clear evidence indicates one of the earliest pre-symptomatic and functional changes associated with ALS occurs distally in the motor neuron axons and at the synaptic terminals (Moloney et al., 2014). In line with this, electrophysiological evidence demonstrates distal changes in excitability are an early hallmark of ALS (Nakata et al., 2006). While it is clear that electrophysiological changes are intimately linked with ALS pathology, the underlying molecular alterations that result in such physiological outcomes remains unknown.

**FIGURE 1:**
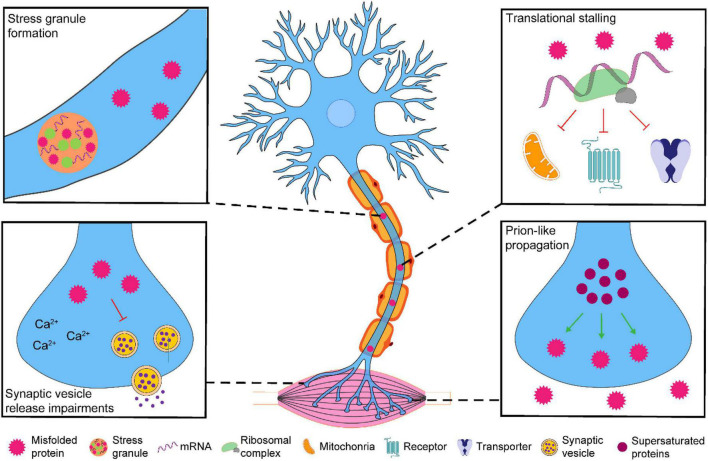
The possible effects of pathological accumulation of ALS-associated proteins within the axo-synaptic compartment of motor neurons. Pathological accumulation of ALS-associated proteins (TDP-43, FUS, C9orf72-associated di-peptide repeats and SOD1) have been observed in the axo-synaptic compartment of motor neurons. The axo-synaptic proteome is supersaturated and conducive to protein misfolding and aggregation that have the capacity to propagate to neighboring cells. The accumulation of these proteins can lead to stress granule formation and translational stalling of key axo-synaptic proteins. Pathological accumulation of ALS-associated proteins can alter Ca^2+^ dynamics and synaptic vesicle release. Collectively, the impairment of these processes may contribute to the synaptic dysfunction associated with ALS, leading to the progressive neurodegeneration of spinal cord motor neurons.

There has been much focus on perturbations of global/somal motor neuron proteostasis and axo-synaptic function in ALS. Despite the axo-synaptic compartment yielding ∼95% of the motor neuron volume (Muzio and Cascella, 2022), little investigation of the proteostasis status within this compartment has been carried out. However, recent landmark studies indicate the presence of a disturbed proteome within the axo-synaptic compartment that can lead to synaptic dysfunction and motor neuron degeneration (Figure 1). Here in this review, we focus on how perturbations to proteome homeostasis within axo-synaptic compartments may contribute to ALS, particularly focusing on critical areas in: (1) understanding the susceptibility of axons and synaptic terminals to misfolded proteins, (2) their perturbed responses to proteostasis disturbances, and (3) how these responses contribute to the synaptic changes observed in ALS.

## Maintaining (sub-) proteome homeostasis in motor neurons

Proteome dyshomeostasis is a common feature of neurodegenerative diseases. The vulnerability of specific cell types within various neurodegenerative disorders can be rationalized to lie inherently within their proteome (Ciryam et al., 2013, 2017; Kundra et al., 2017). Using transcriptomic and proteomic data from healthy spinal cord motor neurons, we demonstrated that ALS-vulnerable spinal cord motor neurons contain many proteins expressed at levels higher than their expected solubility compared to ALS-resistant ocular motor neurons (Yerbury et al., 2019). These proteins are deemed supersaturated and vulnerable to misfolding and aggregation, particularly under conditions of proteostatic stress. A consequence of this supersaturated proteome is that it requires a greater reliance on the proteostasis network of the cell to prevent a collapse. However, previous work indicates motor neurons already have a reduced proteostasis capacity, including reduced mounting of the heat shock response (Batulan et al., 2003) and ubiquitin expression (Brockington et al., 2013), highlighting their vulnerability to proteome dyshomeostasis.

Motor neurons are a uniquely long neuronal sub-type, spanning distances of up to 1 meter, containing a vast proteome. A spinal cord motor neuron can innervate up to 1000 muscle fibers with each synaptic terminal populated by up to 1 million individual protein molecules, creating numerous complex sub-proteomes for the motor neuron to maintain. Whilst many proteins are trafficked from the soma to the synaptic terminal, recent advances in understanding proteostasis with increased granularity demonstrate the synaptic terminal has a local transcriptome and translatome that responds to intra- and -extracellular stimuli (Glock et al., 2021; Perez et al., 2021; Soykan et al., 2021). Traditionally centrifugal isolation has been used to distinguish the transcriptomic and proteomic profiles of soma and axo-synaptic compartments. However, there have been recent technological advances including, the use of soma-axonal culturing chambers, RNA-scope and non-canonical amino acid labeling to provide improved spatiotemporal resolution to dissect differences within sub-compartments (Bai and Witzmann, 2007). Utilization of these techniques have aided in comprehending the difficulties in maintaining proteostasis at the synapse, yet primarily investigate hippocampal pyramidal cells and interneurons. However, these findings may only be extrapolated in spinal cord motor neurons, considering the vast distance between the soma and their unique presynaptic terminals (neuromuscular junction).

The difficulty in maintaining synaptic proteostasis has been postulated as a potential origin of in several neurodegenerative diseases, notably Alzheimer’s and Parkinson’s disease (Freer et al., 2019; Nachman and Verstreken, 2022). Synaptic dysfunction is a common theme across many neurodegenerative diseases and typically occurs early in disease progression. Recent work suggests that many supersaturated proteins are involved in synaptic processes (Freer et al., 2019). Consistent with this, we demonstrated that the sub-proteome of synaptic terminal spinal cord motor neurons is more supersaturated than the entire motor neuron proteome (Lum et al., 2022). This indicates that the synaptic terminal sub-proteome is particularly vulnerable to proteome stress and misfolding, in an already vulnerable proteome. Furthermore, we have found that many genes down-regulated in ALS are enriched in the synapse and encode axo-synaptic proteins integral to their function (Yerbury et al., 2019; Lum et al., 2022). Whilst, proteostasis and synaptic dysfunction are common hallmarks of neurodegenerative diseases, there is a growing focus on investigating proteostasis disturbances within the axo-synaptic compartment, yet many questions remain as to how synaptic proteostasis dysfunction may contribute to disease pathology.

## Evidence of proteome dyshomeostasis at the axon and synapse

### Fused in sarcoma

Fused in sarcoma (FUS) is a nucleic acid-binding protein, playing a role in RNA metabolism. An accumulation of mislocalized cytoplasmic FUS, can be observed in ALS postmortem tissue, where it can form insoluble aggregates (Neumann et al., 2009). Of all the primary ALS-associated aggregation-prone proteins, FUS has the most well-defined role within axons and synaptic terminals. Localization of FUS within RNA granules has been repeatedly reported in the axon, dendrites and pre-and post-synaptic terminals (Sephton and Yu, 2015; Yokoi et al., 2017; Deshpande et al., 2019), including the neuromuscular junction of healthy tissues (So et al., 2018; Picchiarelli et al., 2019). Cortical and hippocampal cultures indicate FUS is localized in both excitatory and inhibitory synapses, where it is preferentially located between the endoplasmic reticulum and synaptic vesicle pools (Sahadevan et al., 2021).

Synaptic FUS primarily localizes to exons and 3′UTRs of RNAs, indicating a role in RNA transport, local translation and stabilization (Sahadevan et al., 2021). In particular, synaptic FUS binds to RNAs encoding many receptors and transporters involved in glutamatergic and GABAergic signaling. Synaptic FUS has been demonstrated to aid in dendritic spine formation and regulate mRNA translation (Fujii et al., 2005), highlighting its importance in synaptic integrity and function. ALS patient-derived motor neurons show an increase in FUS-positive clusters within the axon and synapses. Concurrently, increased Bassoon (pre-synaptic marker) and Homer1 (post-synaptic marker) clusters were also observed within synapses (Deshpande et al., 2019). Given the known roles of FUS at the synapse, these results suggest FUS pathologically accumulates at the synapse in ALS, potentially altering local mRNA control and induce aggregation, collectively perturbing local proteostasis.

Axo-synaptic FUS accumulation has been observed in Fus^Δ^ ^NLS/+^ mice (Scekic-Zahirovic et al., 2017, 2021). Fus^Δ^ ^NLS/+^ mice show an ALS/FTD phenotype with cognitive and motor impairments at 4 and 10 months, respectively, compared to FUS^+/+^ control mice. Axo-synaptic FUS accumulation was associated with altered cortical synaptogenesis, predominantly inhibitory interneurons. Furthermore, six-month old mice showed differential synaptic RNA levels, with a large proportion of genes up-regulated. An enrichment of mRNAs up-regulated were direct binders of FUS; however, a large majority of mRNAs down-regulated were not known synaptic FUS targets, indicating an in-direct mechanism of regulation. Axo-synaptic FUS was associated with increased stability of many genes (enriched in mRNAs containing exonic regions) corresponding to the synaptic-specific function. Additionally, decreased stability (enriched in mRNAs containing 3′UTR) was observed in mRNAs encoding for ribosomal localization, gene expression and translation, processes responsible for maintaining proteostasis. In line with the aforementioned transcriptional changes, protein level alterations were also observed in numerous GABAergic and glutamatergic signaling proteins, indicating that axo-synaptic FUS accumulation subsequently changes the synaptic sub-proteome that likely contributes to synaptogenesis impairments. Whilst, loss of spinal cord motor neurons are observed in the model, synaptic changes of spinal cord motor neurons have not been examined. Considering the number of GABAergic and glutamatergic boutons that extend along spinal cord motor neurons, there is scope to investigate how axo-synaptic FUS accumulation may affect receptor expression and signaling at the post-synaptic terminals that extend along spinal cord motor neurons.

Mutations in the FUS gene are causative of ALS (Kwiatkowski et al., 2009; Vance et al., 2009). Intra-axonal mutant FUS has demonstrated to accumulate in cultured primary neurons derived from m*Fus*^–/–^/hg*FUS*^*R*521*H*^ mice (López-Erauskin et al., 2018). Furthermore, spinal cord RNA expression profiles of m*Fus*^–/–^/hg*FUS*^*R*521*C*^ and m*Fus*^–/–^/hg*FUS*^*R*521*H*^ mice showed a down-regulation of genes encoding for glutamate signaling, in addition to ribosomal proteins and protein translation (López-Erauskin et al., 2018). Similarly, up-regulated genes encode for the eIF2a signaling pathway. eIF2a is a crucial factor for translation initiation and is phosphorylated as part of the integrated stress response (ISR), to stall protein synthesis to alleviate the proteome load (Costa-Mattioli and Walter, 2020). Consistent with this, increased intra-axonal FUS accumulation, pEIF2a and reduced protein synthesis within the sciatic nerve were observed in m*Fus*^–/–^/hg*FUS*^*R*521*C*^ and m*Fus*^–/–^/hg*FUS*^*R*521*H*^ mice compared to wild-type and FUS^WT^ controls (López-Erauskin et al., 2018). Collectively, this work suggests that the accumulation of mutant FUS within axo-synaptic compartments activates the integrated stress response, leading to reduced protein synthesis. Whilst, this study did not examine if a specific set of genes were translationally stalled, it will be important to know how stalling of these genes encode for synaptic functions that may contribute to motor neuron degeneration.

### Transactive response DNA-binding protein-43

Transactive response DNA-binding protein (TDP-43) is a nuclear ribonucleoprotein that binds to UG-rich repeats of target RNAs to regulate gene transcription, mRNA splicing and transport. TDP-43 is predominantly localized to the nucleus, but contains a nuclear export sequence to aid in the nuclear-cytoplasmic shuffling of target RNAs. In healthy neurons, TDP-43 has been shown to localize with axons, synapses and neuromuscular junctions, aiding in RNA transport and stability (Fallini et al., 2012; Narayanan et al., 2013; Ishiguro et al., 2016). Furthermore, examination of axonal TDP-43 suggests subpopulations of TDP-43 RNP pools with different biophysical properties dependent on their distance from to the soma, suggesting diverse physiological roles and aggregation propensity (Gopal et al., 2017).

Postmortem examination of a large majority of ALS cases shows aberrant mislocalization of TDP-43 in the cytoplasm and depletion within the nucleus of motor neurons (Neumann et al., 2009). Altman et al. (2021) recently observed elevated levels of TDP-43 and its pathological phosphorylated form, pTDP-43, in intra-muscular nerves of a small number of sporadic ALS patient biopsies. Furthermore, a more extensive retrospective study of 114 patient biopsies with no history of ALS found biopsies containing axonal pTDP-43 within intra-muscular nerve bundles were later diagnosed with ALS, suggesting diagnostic potential of pTDP-43 in peripheral axons (Kurashige et al., 2022). Furthermore, axonal pTDP-43 has also been observed in post-mortem tissue (Altman et al., 2021; Kurashige et al., 2022) and C9orf72 patient iPSC-derived motor neurons (Altman et al., 2021). Similar findings were reported in intra-muscle axons and even the neuromuscular junction of TDP^ΔNLS^ mice (Altman et al., 2021). Axonal accumulation of pTDP-43 was shown to colocalize with the ribonucleoprotein component, G3BP1 and RNAs, indicating the formation of ribonucleoprotein (RNP) condensates within axons. The formation of RNP condensates within the soma represses RNA translation and is believed to be a compensatory mechanism to alleviate the misfolded proteome load in ALS. In line with this, TDP-43 mislocalization was associated with reduced protein synthesis within the axons and presynaptic terminals of C9orf72 iPSC-derived neurons, primary neuromuscular co-cultures and TDP^ΔNLS^ mice (Altman et al., 2021). Proteomic analysis of axoplasmic lysates from TDP^ΔNLS^ mice found a reduction in nuclear-encoded mitochondrial proteins, including ATP5A1, Cox4i1 and Ndufa4, despite showing modest increases in mRNA abundance, suggesting axonal TDP-43 containing RNPs sequester and impair the local translation of these mitochondrial transcripts. Furthermore, it was demonstrated that this impairment of local synaptic and mitochondrial protein synthesis led to reduced neuromuscular junction function and neurodegeneration (Altman et al., 2021). This work not only highlights that aggregation-prone proteins such as TDP-43 pathologically accumulate in the axo-synaptic compartments, but that their presence within these compartments can have detrimental consequences that can lead to neurodegeneration.

### C9orf72

Abnormal expansion of GGGGCC hexanucleotide repeats within the *C9orf72* gene is the most frequent genetic association with ALS. It has been proposed that GGGGCC expansion repeats may play a pathogenic role through several mechanisms, including loss of C9orf72 expression and function. C9orf72 protein has been shown to localize to the pre-synaptic terminals, where it interacts with the RAB3 family of proteins involved with synaptic vesicle release (Atkinson et al., 2015; Frick et al., 2018). Furthermore, GGGGCC hexanucleotide repeats within the *C9orf72* gene can generate the synthesis of di-peptide repeat (DPRs) species produced by repeat-associated non-AUG (RAN) translation (Mori et al., 2013). In C9orf72 cases, the production of DPR species has been shown to disrupt nuclear-cytoplasmic transport and mislocalization of TDP-43 (Solomon et al., 2018). As previously discussed, C9orf72 mutations have shown to produce axo-synaptic accumulation of pTDP-43 and synaptic dysfunction (Altman et al., 2021), suggesting an indirect role of DPRs on axo-synaptic proteostasis.

In addition to the in-direct role DPRs have on axo-synaptic proteostasis, they may also have a more local and direct role. There are five DPRs produced from sense and antisense (poly-GA, poly-GR, poly-GP, poly-PR and poly-PA). The contribution of each individual DPR species is still unclear. Poly-GA are the most abundant within cytoplasmic inclusions. Furthermore, poly-GA has shown to be present within dystrophic neurites of the cortex, but not in the spinal cord of ALS cases (Mackenzie et al., 2013). Poly-GA has been shown to be present in the neurites GA mouse model (Jensen et al., 2020). In primary motor neuron cultures, GA aggregates were mobile and inversely correlated with GA repeat length. Furthermore, neurons containing GA aggregates showed a reduction in synaptic vesicle release that was associated with a reduction in the synaptic vesicle protein, SV2, as a consequence of GA aggregates sequestering SV2 mRNA. Furthermore, a similar decrease in SV2 mRNA and protein expression was observed in C9orf72 iPSC-derived neurons. However, up-regulation of SV2 was able to reverse synaptic vesicle release impairments (Jensen et al., 2020). Whilst it is unclear if other DPRs show similar localization and effects, this study highlights the potential of targeting synaptic function as a potential therapeutic avenue for ALS-associated axo-synaptic proteostasis imbalance.

### Superoxide dismutase-1

There have been >160 mutations identified within the *superoxide dismutase-1* (*SOD1*) gene associated with ALS. Unlike FUS and TDP-43, SOD1 is not an RNA-binding protein and is an antioxidant enzyme that functions to catalyze superoxide free radicals to molecular oxygen or hydrogen peroxide. However, similarly SOD1 inclusions form in the cytoplasm of motor neurons in familial ALS cases (Shibata et al., 1996). Although misfolded SOD1 also been suggested to be associated with non-SOD1 familial- and sporadic-ALS cases (Guareschi et al., 2012; Grad et al., 2014; Tokuda et al., 2019; Trist et al., 2022). SOD1 is primarily localized to the cytoplasm and mitochondria. However, it can also be found in the nucleus (Gertz et al., 2012). To our knowledge, it has yet to be confirmed if SOD1 is present in the axons or synaptic terminals. Although, transcriptomic and proteomic analyses indicate that SOD1 mRNA and protein are localized and translated within synaptic terminals, including the neuromuscular junction (Ketterer et al., 2010; Glock et al., 2021).

There have been several SOD1 mouse models developed over the last few decades. However, none is better characterized than the SOD1^G93A^ model. SOD1^G93A^ mice show spatiotemporal synaptic impairments, which have been previously summarized in detail (Fogarty, 2019). SOD1 aggregates have been extensively reported in spinal cord motor neurons of SOD1^G93A^ mice (Deng et al., 2006; Gill et al., 2019). However, evidence for their axo-synaptic localization is limited. Immunoblots of sciatic nerves have shown mutant SOD1 aggregates (Turner et al., 2003), indicating their presence in axo-synaptic compartments. In line with this, mutant SOD1 accumulation has been observed in ventral roots as early as four weeks of age, before their presence within the ventral horn and onset of motor impairment (Shiraishi et al., 2021). This indicates mutant SOD1 may accumulate in a distal-proximal fashion. However, it has yet to be investigated if SOD1 aggregates are located more peripherally, such as in the neuromuscular junction of motor neurons.

In support of the distal-proximal accumulation of SOD1, sciatic nerve inoculation of spinal homogenates from paralyzed SOD1 mutant mice in SOD1^G85R:YFP^ mice produces motor deficits and motor neuron pathology (Ayers et al., 2014, 2016). The formation of SOD1^G85R:YFP^ inclusions pathology are initially observed in the ipsilateral DRG and follow a retrograde trajectory along neuroanatomical tracts toward the brainstem (Ayers et al., 2016). The route of SOD1 inclusions in this model along neuroanatomical tracts is suggestive of trans-synaptic prion-like propagation spread and is supported by the observation of SOD1 inclusions within the neuropil of spinal cord and brainstem (Ayers et al., 2016). In line with the spatiotemporal spread of ALS symptom progression, there is evidence to suggest that the primary aggregation prone proteins (SOD1, TDP-43 and FUS) are capable of forming prion-like seeds and propagating, potentially *via* trans-synaptic pathways (McAlary et al., 2020). Considering that the motor neuron synapse is supersaturated, it may provide an environment conducive for spreading of misfolded protein conformations.

Whilst there has been limited investigation if aggregated SOD1 is present within axo-synaptic compartments, mutant SOD1 has shown to disrupt synaptic function. Presynaptic infusion of mutant SOD1^G85R^ or SOD1^H46R^ protein in the squid giant synapse has been demonstrated to inhibit anterograde transport, whilst an infusion of SOD1^WT^ only showed modest impairments (Bosco et al., 2010; Song et al., 2013). Furthermore, infusion of SOD1^G85R^ has shown to rapidly impair synaptic vesicle availability and release, which slowly returns to normal following the passive diffusion of SOD1^G85R^ out of the presynaptic terminal (Song, 2020). SOD1^G85R^-induced vesicle release impairments were shown to be mediated *via* synaptic and peri-synaptic Ca^2+^ levels and localization alterations. However, the mechanistic links that caused these changes are still, but may be necessary for identifying intervention targets. Collectively, these studies demonstrate that the presence of synaptic mutant SOD1 can disrupt synaptic function. However, the molecular mechanisms that lead to these changes are still unclear. Therefore, it is unclear what contribution potential axo-synaptic pathological aggregates have on the numerous reports of synaptic dysfunction in this model. Furthermore, evidence indicates axo-synaptic FUS and TDP-43 accumulation may exert their pathological effects *via* their RNA binding targets. However, SOD1 does not bind RNA and therefore may represent a more “pure” system of how proteostasis collapse leads to synaptic dysfunction.

## Concluding remarks

There is now established evidence that ALS-associated proteins have a pathological role in the axo-synaptic compartment that may contribute to disease pathology. Whilst, the supersaturated environment of the synapse indicates it may be a more conducive environment for proteins to misfold, it is still not clear if this process begins in the synapse or if they are trafficked from the soma. Based on this initial body of evidence that ALS-associated proteins can pathologically accumulate in the axo-synaptic compartment, understanding if the synaptic excitability changes observed in ALS are caused by more local proteostasis alterations warrants investigation. Whilst the currently available treatments for ALS, riluzole and edaravone, have shown to work in part by improving synaptic function, their limited efficacy may be due to their inability to modulate toxic protein species (Lamanauskas and Nistri, 2008; Jaiswal, 2016; Li et al., 2022). Preclinical and clinical trials using anti-sense oligonucleotides are on-going with encouraging results (Amado and Davidson, 2021). Whilst, these will fail to directly remove aggregates already localized to the soma and axo-synaptic compartment, they may ease the continual axo-synaptic accumulation of toxic species and proteostasis load. In addition, there is promising work investigating the potential of proteolysis- and autophagy-targeting chimeras to facilitate the removal of toxic protein species (Schmidt et al., 2021), however, to our knowledge it remains unclear if these are able to transported/synthesized within the axo-synaptic compartment. It is likely that the best therapeutic avenue will need to be multi-modal and remove toxic species and restore axo-synaptic proteostasis and function. Proteostasis encapsulates the synthesis, maturation, transport and degradation of the proteome, therefore understanding each aspect of proteostasis within the axo-synaptic compartment and how these are perturbed in ALS is vital. Importantly, dissecting the role of pathological accumulation of proteins within the soma and axo-synaptic compartment and the local and global responses may lead to identifying more targeting points of therapeutic intervention.

## Author contributions

JL and JY conceptualized, directed, and wrote and edited the manuscript. Both authors contributed to the article and approved the submitted version.

## References

[B1] Altman T. Ionescu A. Ibraheem A. Priesmann D. Gradus-Pery T. Farberov L. (2021). Axonal TDP-43 condensates drive neuromuscular junction disruption through inhibition of local synthesis of nuclear encoded mitochondrial proteins. *Nat. Commun.* 12:6914. 10.1038/s41467-021-27221-8 34824257 PMC8617040

[B2] Amado D. A. Davidson B. L. (2021). Gene therapy for ALS: A review. *Mol. Ther.* 29 3345–3358. 10.1016/j.ymthe.2021.04.008 33839324 PMC8636154

[B3] Atkinson R. A. K. Fernandez-Martos C. M. Atkin J. D. Vickers J. C. King A. E. (2015). C9ORF72 expression and cellular localization over mouse development. *Acta Neuropathol. Commun.* 3:59. 10.1186/s40478-015-0238-7 26408000 PMC4582620

[B4] Ayers J. I. Fromholt S. E. O’Neal V. M. Diamond J. H. Borchelt D. R. (2016). Prion-like propagation of mutant SOD1 misfolding and motor neuron disease spread along neuroanatomical pathways. *Acta Neuropathol.* 131 103–114. 10.1007/s00401-015-1514-0 26650262 PMC4699876

[B5] Ayers J. I. Fromholt S. Koch M. DeBosier A. McMahon B. Xu G. (2014). Experimental transmissibility of mutant SOD1 motor neuron disease. *Acta Neuropathol.* 128 791–803. 10.1007/s00401-014-1342-7 25262000

[B6] Bai F. Witzmann F. A. (2007). Synaptosome proteomics. *Subcell Biochem.* 43 77–98. 17953392 10.1007/978-1-4020-5943-8_6 PMC2853956

[B7] Batulan Z. Shinder G. A. Minotti S. He B. P. Doroudchi M. M. Nalbantoglu J. (2003). High threshold for induction of the stress response in motor neurons is associated with failure to activate HSF1. *J. Neurosci.* 23 5789–5798. 10.1523/JNEUROSCI.23-13-05789.2003 12843283 PMC6741252

[B8] Bosco D. A. Morfini G. Karabacak N. M. Song Y. Gros-Louis F. Pasinelli P. (2010). Wild-type and mutant SOD1 share an aberrant conformation and a common pathogenic pathway in ALS. *Nat. Neurosci.* 13 1396–1403. 10.1038/nn.2660 20953194 PMC2967729

[B9] Brockington A. Ning K. Heath P. R. Wood E. Kirby J. Fusi N. (2013). Unravelling the enigma of selective vulnerability in neurodegeneration: Motor neurons resistant to degeneration in ALS show distinct gene expression characteristics and decreased susceptibility to excitotoxicity. *Acta Neuropathol.* 125 95–109. 10.1007/s00401-012-1058-5 23143228 PMC3535376

[B10] Ciryam P. Lambert-Smith I. A. Bean D. M. Freer R. Cid F. Tartaglia G. G. (2017). Spinal motor neuron protein supersaturation patterns are associated with inclusion body formation in ALS. *Proc. Natl. Acad. Sci. U.S.A.* 114 E3935–E3943. 10.1073/pnas.1613854114 28396410 PMC5441770

[B11] Ciryam P. Tartaglia G. G. Morimoto R. I. Dobson C. M. Vendruscolo M. (2013). Widespread aggregation and neurodegenerative diseases are associated with supersaturated proteins. *Cell Rep.* 5 781–790. 10.1016/j.celrep.2013.09.043 24183671 PMC3883113

[B12] Costa-Mattioli M. Walter P. (2020). The integrated stress response: From mechanism to disease. *Science* 368:eaat5314. 10.1126/science.aat5314 32327570 PMC8997189

[B13] Deng H.-X. Shi Y. Furukawa Y. Zhai H. Fu R. Liu E. (2006). Conversion to the amyotrophic lateral sclerosis phenotype is associated with intermolecular linked insoluble aggregates of SOD1 in mitochondria. *Proc. Natl. Acad. Sci. U.S.A.* 103 7142–7147. 10.1073/pnas.0602046103 16636275 PMC1447523

[B14] Deshpande D. Higelin J. Schoen M. Vomhof T. Boeckers T. M. Demestre M. (2019). Synaptic FUS localization during motoneuron development and its accumulation in human ALS synapses. *Front. Cell Neurosci.* 13:256. 10.3389/fncel.2019.00256 31244613 PMC6582137

[B15] Fallini C. Bassell G. J. Rossoll W. (2012). The ALS disease protein TDP-43 is actively transported in motor neuron axons and regulates axon outgrowth. *Hum. Mol. Genet.* 21 3703–3718. 10.1093/hmg/dds205 22641816 PMC3406762

[B16] Fogarty M. J. (2019). Amyotrophic lateral sclerosis as a synaptopathy. *Neural Regen. Res.* 14 189–192. 10.4103/1673-5374.244782 30530995 PMC6301167

[B17] Freer R. Sormanni P. Ciryam P. Rammner B. Rizzoli S. O. Dobson C. M. (2019). Supersaturated proteins are enriched at synapses and underlie cell and tissue vulnerability in Alzheimer’s disease. *Heliyon* 5:e02589. 10.1016/j.heliyon.2019.e02589 31768427 PMC6872855

[B18] Frick P. Sellier C. Mackenzie I. R. A. Cheng C.-Y. Tahraoui-Bories J. Martinat C. (2018). Novel antibodies reveal presynaptic localization of C9orf72 protein and reduced protein levels in C9orf72 mutation carriers. *Acta Neuropathol. Commun.* 6:72. 10.1186/s40478-018-0579-0 30075745 PMC6091050

[B19] Fujii R. Okabe S. Urushido T. Inoue K. Yoshimura A. Tachibana T. (2005). The RNA binding protein TLS is translocated to dendritic spines by mGluR5 activation and regulates spine morphology. *Curr. Biol.* 15 587–593. 10.1016/j.cub.2005.01.058 15797031

[B20] Gertz B. Wong M. Martin L. J. (2012). Nuclear localization of human SOD1 and mutant SOD1-specific disruption of survival motor neuron protein complex in transgenic amyotrophic lateral sclerosis mice. *Journal of Neuropathol. Exp. Neurol.* 71 162–177. 10.1097/NEN.0b013e318244b635 22249462 PMC3432922

[B21] Gill C. Phelan J. P. Hatzipetros T. Kidd J. D. Tassinari V. R. Levine B. (2019). SOD1-positive aggregate accumulation in the CNS predicts slower disease progression and increased longevity in a mutant SOD1 mouse model of ALS. *Sci. Rep.* 9:6724. 10.1038/s41598-019-43164-z 31040321 PMC6491559

[B22] Glock C. Biever A. Tushev G. Nassim-Assir B. Kao A. Bartnik I. (2021). The translatome of neuronal cell bodies, dendrites, and axons. *Proc. Natl. Acad. Sci. U.S.A.* 118:e2113929118. 10.1073/pnas.2113929118 34670838 PMC8639352

[B23] Gopal P. P. Nirschl J. J. Klinman E. Holzbaur E. L. F. (2017). Amyotrophic lateral sclerosis-linked mutations increase the viscosity of liquid-like TDP-43 RNP granules in neurons. *Proc. Natl. Acad. Sci. U.S.A.* 114 E2466–E2475. 10.1073/pnas.1614462114 28265061 PMC5373408

[B24] Grad L. I. Yerbury J. J. Turner B. J. Guest W. C. Pokrishevsky E. O’Neill M. A. (2014). Intercellular propagated misfolding of wild-type Cu/Zn superoxide dismutase occurs *via* exosome-dependent and -independent mechanisms. *Proc. Natl. Acad. Sci. U.S.A.* 111 3620–3625. 10.1073/pnas.1312245111 24550511 PMC3948312

[B25] Guareschi S. Cova E. Cereda C. Ceroni M. Donetti E. Bosco D. A. (2012). An over-oxidized form of superoxide dismutase found in sporadic amyotrophic lateral sclerosis with bulbar onset shares a toxic mechanism with mutant SOD1. *Proc. Natl. Acad. Sci. U.S.A.* 109 5074–5079. 10.1073/pnas.1115402109 22416121 PMC3324021

[B26] Ishiguro A. Kimura N. Watanabe Y. Watanabe S. Ishihama A. (2016). TDP-43 binds and transports G-quadruplex-containing mRNAs into neurites for local translation. *Genes Cells* 21 466–481. 10.1111/gtc.12352 26915990

[B27] Jaiswal M. K. (2016). Riluzole but not melatonin ameliorates acute motor neuron degeneration and moderately inhibits SOD1-mediated excitotoxicity induced disrupted mitochondrial Ca2+ signaling in amyotrophic lateral sclerosis. *Front. Cell. Neurosci.* 10:295. 10.3389/fncel.2016.00295 28111541 PMC5216043

[B28] Jensen B. K. Schuldi M. H. McAvoy K. Russell K. A. Boehringer A. Curran B. M. (2020). Synaptic dysfunction induced by glycine-alanine dipeptides in C9orf72-ALS/FTD is rescued by SV2 replenishment. *EMBO Mol. Med.* 12:e10722. 10.15252/emmm.201910722 32347002 PMC7207170

[B29] Ketterer C. Zeiger U. Budak M. T. Rubinstein N. A. Khurana T. S. (2010). Identification of the neuromuscular junction transcriptome of extraocular muscle by laser capture microdissection. *Invest. Ophthalmol. Vis. Sci.* 51 4589–4599. 10.1167/iovs.09-4893 20393109 PMC2941174

[B30] Knowles T. P. J. Vendruscolo M. Dobson C. M. (2014). The amyloid state and its association with protein misfolding diseases. *Nat. Rev. Mol. Cell Biol.* 15 384–396. 10.1038/nrm3810 24854788

[B31] Kundra R. Ciryam P. Morimoto R. I. Dobson C. M. Vendruscolo M. (2017). Protein homeostasis of a metastable subproteome associated with Alzheimer’s disease. *Proc. Natl. Acad. Sci. U.S.A.* 114 E5703–E5711. 10.1073/pnas.1618417114 28652376 PMC5514703

[B32] Kundra R. Dobson C. M. Vendruscolo M. (2020). A cell- and tissue-specific weakness of the protein homeostasis system underlies brain vulnerability to protein aggregation. *iScience* 23:100934. 10.1016/j.isci.2020.100934 32146327 PMC7063235

[B33] Kurashige T. Morino H. Murao T. Izumi Y. Sugiura T. Kuraoka K. (2022). TDP-43 accumulation within intramuscular nerve bundles of patients with amyotrophic lateral sclerosis. *JAMA Neurol.* 79 693–701. 10.1001/jamaneurol.2022.1113 35604654 PMC9127711

[B34] Kwiatkowski T. J. Bosco D. A. LeClerc A. L. Tamrazian E. Vanderburg C. R. Russ C. (2009). Mutations in the FUS/TLS gene on chromosome 16 cause familial amyotrophic lateral sclerosis. *Science* 323 1205–1208. 10.1126/science.1166066 19251627

[B35] Lamanauskas N. Nistri A. (2008). Riluzole blocks persistent Na+ and Ca2+ currents and modulates release of glutamate *via* presynaptic NMDA receptors on neonatal rat hypoglossal motoneurons *in vitro*. *Eur. J. Neurosci.* 27 2501–2514. 10.1111/j.1460-9568.2008.06211.x 18445055

[B36] Leigh P. N. Whitwell H. Garofalo O. Buller J. Swash M. Martin J. E. (1991). Ubiquitin-immunoreactive intraneuronal inclusions in amyotrophic lateral sclerosis. Morphology, distribution, and specificity. *Brain* 114(Pt. 2), 775–788. 10.1093/brain/114.2.775 1646064

[B37] Li Q. Feng Y. Xue Y. Zhan X. Fu Y. Gui G. (2022). Edaravone activates the GDNF/RET neurotrophic signaling pathway and protects mRNA-induced motor neurons from iPS cells. *Mol. Neurodegen.* 17:8. 10.1186/s13024-021-00510-y 35012575 PMC8751314

[B38] López-Erauskin J. Tadokoro T. Baughn M. W. Myers B. McAlonis-Downes M. Chillon-Marinas C. (2018). ALS/FTD-linked mutation in FUS Suppresses intra-axonal protein synthesis and drives disease without nuclear loss-of-function of FUS. *Neuron* 100 816–830.e7. 10.1016/j.neuron.2018.09.044 30344044 PMC6277851

[B39] Lum J. S. Berg T. Chisholm C. G. Vendruscolo M. Yerbury J. J. (2022). Vulnerability of the spinal motor neuron presynaptic terminal sub-proteome in ALS. *Neurosci. Lett.* 778:136614. 10.1016/j.neulet.2022.136614 35367314

[B40] Mackenzie I. R. Arzberger T. Kremmer E. Troost D. Lorenzl S. Mori K. (2013). Dipeptide repeat protein pathology in C9ORF72 mutation cases: Clinico-pathological correlations. *Acta Neuropathol.* 126 859–879. 10.1007/s00401-013-1181-y 24096617

[B41] McAlary L. Chew Y. L. Lum J. S. Geraghty N. J. Yerbury J. J. Cashman N. R. (2020). Amyotrophic lateral sclerosis: Proteins, proteostasis, prions, and promises. *Front. Cell. Neurosci.* 14:581907. 10.3389/fncel.2020.581907 33328890 PMC7671971

[B42] Moloney E. B. de Winter F. Verhaagen J. (2014). ALS as a distal axonopathy: Molecular mechanisms affecting neuromuscular junction stability in the presymptomatic stages of the disease. *Front. Neurosci.* 8:252. 10.3389/fnins.2014.00252 25177267 PMC4132373

[B43] Mori K. Weng S.-M. Arzberger T. May S. Rentzsch K. Kremmer E. (2013). The C9orf72 GGGGCC repeat is translated into aggregating dipeptide-repeat proteins in FTLD/ALS. *Science* 339 1335–1338. 10.1126/science.1232927 23393093

[B44] Muzio M. R. Cascella M. (2022). “Histology, axon,” in *StatPearls*, (Treasure Island, FL: StatPearls Publishing). Available online at: http://www.ncbi.nlm.nih.gov/books/NBK554388/ (accessed June 5, 2022) 32119275

[B45] Nachman E. Verstreken P. (2022). Synaptic proteostasis in Parkinson’s disease. *Curr. Opin. Neurobiol.* 72 72–79. 10.1016/j.conb.2021.09.001 34653835

[B46] Nakata M. Kuwabara S. Kanai K. Misawa S. Tamura N. Sawai S. (2006). Distal excitability changes in motor axons in amyotrophic lateral sclerosis. *Clin. Neurophysiol.* 117 1444–1448. 10.1016/j.clinph.2006.04.005 16765084

[B47] Narayanan R. K. Mangelsdorf M. Panwar A. Butler T. J. Noakes P. G. Wallace R. H. (2013). Identification of RNA bound to the TDP-43 ribonucleoprotein complex in the adult mouse brain. *Amyotroph. Lateral Scler. Frontotemporal Degener.* 14 252–260. 10.3109/21678421.2012.734520 23134510

[B48] Neumann M. Rademakers R. Roeber S. Baker M. Kretzschmar H. A. Mackenzie I. R. A. (2009). A new subtype of frontotemporal lobar degeneration with FUS pathology. *Brain* 132 2922–2931. 10.1093/brain/awp214 19674978 PMC2768659

[B49] Perez J. D. Fusco C. M. Schuman E. M. (2021). A functional dissection of the mRNA and locally synthesized protein population in neuronal dendrites and axons. *Annu. Rev. Genet.* 55 183–207. 10.1146/annurev-genet-030321-054851 34460296

[B50] Picchiarelli G. Demestre M. Zuko A. Been M. Higelin J. Dieterlé S. (2019). FUS-mediated regulation of acetylcholine receptor transcription at neuromuscular junctions is compromised in amyotrophic lateral sclerosis. *Nat. Neurosci.* 22 1793–1805. 10.1038/s41593-019-0498-9 31591561 PMC6858880

[B51] Saberi S. Stauffer J. E. Schulte D. J. Ravits J. (2015). Neuropathology of amyotrophic lateral sclerosis and its variants. *Neurol. Clin.* 33 855–876. 10.1016/j.ncl.2015.07.012 26515626 PMC4628785

[B52] Sahadevan S. Hembach K. M. Tantardini E. Pérez-Berlanga M. Hruska-Plochan M. Megat S. (2021). Synaptic FUS accumulation triggers early misregulation of synaptic RNAs in a mouse model of ALS. *Nat. Commun.* 12:3027. 10.1038/s41467-021-23188-8 34021139 PMC8140117

[B53] Scekic-Zahirovic J. Oussini H. E. Mersmann S. Drenner K. Wagner M. Sun Y. (2017). Motor neuron intrinsic and extrinsic mechanisms contribute to the pathogenesis of FUS-associated amyotrophic lateral sclerosis. *Acta Neuropathol.* 133 887–906. 10.1007/s00401-017-1687-9 28243725 PMC5427169

[B54] Scekic-Zahirovic J. Sanjuan-Ruiz I. Kan V. Megat S. De Rossi P. Dieterlé S. (2021). Cytoplasmic FUS triggers early behavioral alterations linked to cortical neuronal hyperactivity and inhibitory synaptic defects. *Nat. Commun.* 12:3028. 10.1038/s41467-021-23187-9 34021132 PMC8140148

[B55] Schmidt M. F. Gan Z. Y. Komander D. Dewson G. (2021). Ubiquitin signalling in neurodegeneration: Mechanisms and therapeutic opportunities. *Cell Death Differ*. 28 570–590. 10.1038/s41418-020-00706-7 33414510 PMC7862249

[B56] Sephton C. F. Yu G. (2015). The function of RNA-binding proteins at the synapse: Implications for neurodegeneration. *Cell. Mol. Life Sci.* 72 3621–3635. 10.1007/s00018-015-1943-x 26047658 PMC4565867

[B57] Shibata N. Hirano A. Kobayashi M. Siddique T. Deng H.-X. Hung W.-Y. (1996). Intense superoxide dismutase-1 immunoreactivity in intracytoplasmic hyaline inclusions of familial amyotrophic lateral sclerosis with posterior column involvement. *J. Neuropathol. Exp. Neurol.* 55 481–490. 10.1097/00005072-199604000-00011 8786408

[B58] Shiraishi W. Yamasaki R. Hashimoto Y. Ko S. Kobayakawa Y. Isobe N. (2021). Clearance of peripheral nerve misfolded mutant protein by infiltrated macrophages correlates with motor neuron disease progression. *Sci. Rep.* 11:16438. 10.1038/s41598-021-96064-6 34385589 PMC8360983

[B59] So E. Mitchell J. C. Memmi C. Chennell G. Vizcay-Barrena G. Allison L. (2018). Mitochondrial abnormalities and disruption of the neuromuscular junction precede the clinical phenotype and motor neuron loss in hFUSWT transgenic mice. *Hum. Mol. Genet.* 27 463–474. 10.1093/hmg/ddx415 29194538 PMC5886082

[B60] Solomon D. A. Stepto A. Au W. H. Adachi Y. Diaper D. C. Hall R. (2018). A feedback loop between dipeptide-repeat protein, TDP-43 and karyopherin-α mediates C9orf72-related neurodegeneration. *Brain* 141 2908–2924. 10.1093/brain/awy241 30239641 PMC6158706

[B61] Song Y. (2020). Synaptic actions of amyotrophic lateral sclerosis-associated G85R-SOD1 in the squid giant synapse. *eNeuro* 7:ENEURO.369–ENEURO.319. 10.1523/ENEURO.0369-19.2020 32188708 PMC7177748

[B62] Song Y. Nagy M. Ni W. Tyagi N. K. Fenton W. A. López-Giráldez F. (2013). Molecular chaperone Hsp110 rescues a vesicle transport defect produced by an ALS-associated mutant SOD1 protein in squid axoplasm. *Proc. Natl. Acad. Sci. U.S.A.* 110 5428–5433. 10.1073/pnas.1303279110 23509252 PMC3619309

[B63] Soykan T. Haucke V. Kuijpers M. (2021). Mechanism of synaptic protein turnover and its regulation by neuronal activity. *Curr. Opin. Neurobiol.* 69 76–83. 10.1016/j.conb.2021.02.006 33744822

[B64] Ticozzi N. Ratti A. Silani V. (2010). Protein aggregation and defective RNA metabolism as mechanisms for motor neuron damage. *CNS Neurol. Disord. Drug Targets* 9 285–296. 10.2174/187152710791292585 20406182

[B65] Tokuda E. Takei Y. Ohara S. Fujiwara N. Hozumi I. Furukawa Y. (2019). Wild-type Cu/Zn-superoxide dismutase is misfolded in cerebrospinal fluid of sporadic amyotrophic lateral sclerosis. *Mol. Neurodegener.* 14:42. 10.1186/s13024-019-0341-5 31744522 PMC6862823

[B66] Trist B. G. Genoud S. Roudeau S. Rookyard A. Abdeen A. Cottam V. (2022). Altered SOD1 maturation and post-translational modification in amyotrophic lateral sclerosis spinal cord. *Brain* awac165. [Epub ahead of print]. 10.1093/brain/awac165 35512359 PMC9473357

[B67] Turner B. J. Lopes E. C. Cheema S. S. (2003). Neuromuscular accumulation of mutant superoxide dismutase 1 aggregates in a transgenic mouse model of familial amyotrophic lateral sclerosis. *Neurosci. Lett.* 350 132–136. 10.1016/S0304-3940(03)00893-0 12972170

[B68] Vance C. Rogelj B. Hortobágyi T. De Vos K. J. Nishimura A. L. Sreedharan J. (2009). Mutations in FUS, an RNA processing protein, cause familial amyotrophic lateral sclerosis type 6. *Science* 323 1208–1211. 10.1126/science.1165942 19251628 PMC4516382

[B69] Yerbury J. J. Ooi L. Blair I. P. Ciryam P. Dobson C. M. Vendruscolo M. (2019). The metastability of the proteome of spinal motor neurons underlies their selective vulnerability in ALS. *Neurosci. Lett.* 704 89–94. 10.1016/j.neulet.2019.04.001 30953736

[B70] Yerbury J. J. Ooi L. Dillin A. Saunders D. N. Hatters D. M. Beart P. M. (2016). Walking the tightrope: Proteostasis and neurodegenerative disease. *J. Neurochem.* 137 489–505. 10.1111/jnc.13575 26872075

[B71] Yokoi S. Udagawa T. Fujioka Y. Honda D. Okado H. Watanabe H. (2017). 3′UTR length-dependent control of SynGAP isoform α2 mRNA by FUS and ELAV-like proteins promotes dendritic spine maturation and cognitive function. *Cell Rep.* 20 3071–3084. 10.1016/j.celrep.2017.08.100 28954225

